# Supplementary material for: BOLD signal response in primary visual cortex to flickering checkerboard increases with stimulus temporal frequency in older adults
Source: PLoS One. 2021 Nov 4;16(11):e0259243. doi: 10.1371/journal.pone.0259243 (PMC8568270; doi:10.1371/journal.pone.0259243)
Supplement: Attachment — Submitted filename: Response_To_Reviewers20210518.docx [file pone.0259243.s004.docx]

**Response to Reviewers**

**We thank the editorial board and reviewers for their very helpful comments. Our manuscript has been revised according to the comments. We think that the comments have improved our manuscript. Our point-to-point responses to each comment were written in bold letters.**

**Responses to Reviewer #1:**

This study is an analysis of BOLD response in primary visual cortex during basic visual processing in a sample of older adults. Prior literature is mixed on whether or not older adults show increased BOLD response in response to increasing visual frequency rates, and the authors aim to test this by using surface-based analyses which may be more sensitive to anatomical specifics of primary visual cortex. The paper is well written and the analyses straightforward. My comments primarily regard clarification on some of the methods/analysis and suggestions to explore potential individual difference effects.

Comment 1-1. Introduction: The authors do a good job outlining the inconsistent findings from previous studies and possible reasons for the inconsistencies. I think the last paragraph of the Intro would also benefit from more explicit statements about how their study design/methods (e.g., surface based analyses) can help to resolve prior inconsistent findings.

**Response: Thank you for pointing this out. A statement of the advantage of the surface-based analysis was added to the last paragraph of the Introduction.**

**“The surface-based analysis will reveal the V1 region related to a temporal frequency change of visual stimuli more accurately than the voxel-based analysis used in previous studies.”**

Comment 1-2. Methods: In the discussion the authors mention the task was completely passive viewing, but were any study procedures implemented to ensure that participants remained awake/had their eyes open during the checkerboard task? A few examples of what I mean: eye-tracking, or a camera positions on the participant’s eyes that the experimenter could monitor, or a post-scan debrief asking if the participant remained awake?

**Response: Experimenters monitored participants’ eyes with a camera toward the eyes through a mirror during the checkerboard task. We added a statement concerning this monitoring in the imaging setup and fMRI procedure section.**

Comment 1-3. Methods: FreeSurfer’s segmentation of the cortical surface can sometimes be problematic, especially at the back of the head where dura is frequently included within the surface boundary of posterior occipital regions. Given the importance of this surfaced-based analysis, where the FreeSurfer surfaces checked or edited to ensure no dura matter was included in the primary regions of interest?

**Response: Thank you for pointing this out. We checked the skull-stripped images, and found surface boundary images (i.e., pial images) included the dura mater. Thus, we removed the dura mater from the pial images by editing brain mask images. This change affected all of the results of the previous manuscript. Thus, figures (Figs 1–4.), tables (Tables 1–2.), and statistical values have been revised in the Results section and supplementary materials. However, the change does not affect the manuscript’s conclusion. Moreover, we added the procedure of removing the dura mater to the Preprocessing section.**

Comment 1-4. Methods: For the first-level individual level analysis, why did the authors choose to model each frequency separately (i.e., three regressors) rather than having one parametric regressor that modeled the different levels of frequency (2,4,8 as weights)? A first-level parametric regressor may be more appropriate if the authors anticipate that the change in BOLD response in response to change in frequency was a dose-response type effect (e.g., BOLD showed a greater increase from 4hz to 8hz compared to 2hz to 4hz).

**Response: Thank you for pointing this out. Parametric fMRI analysis has been conducted, and we added the statement of the analysis to Materials and Methods, Results, and Discussion. The results support our conclusion more clearly than those in the previous manuscript.**

Comment 1-5. Results: Although there is a group effect of increasing frequency being associated with increased BOLD response, only 16 out of 29 participants showed this pattern at the individual level (for at least one hemisphere). Where there any characteristics of the participants that did not show this effect that might explain this (ie., Were they the oldest participants? did they have worse vision? Were they less attentive during scanning?). It would be quite interesting if the authors could quantify the amount of BOLD increase in response to frequency (perhaps through a first-level parametric regressor as suggested above) and link it to individual difference measures (e.g., visual acuity, visual speed of processing).

**Response: We collected individual data of age, MMSE, and visual speed of processing (UFOV) and analyzed the relation with the BOLD responses. However, we could not find a significant relation and did not include the results in the previous manuscript. According to the above comment, the relation between the individual data and the slope coefficient of BOLD slope in the V1 ROI was added, which we obtained using the parametric analysis in Table 4. We added a statement on the exploring to the Introduction, Materials and Methods, Results, and Discussion sections.**

Comment 1-6. Discussion: Given that the authors find this increase in BOLD in response to increasing frequency in older adults (as is found in younger adults), might this suggest that the difficulties older adults have with visual stimuli are occurring further along the visual processing stream in higher level visual areas? Or perhaps top-down processes are interfering with visual processing? As stated above, given the somewhat large individual differences in how many older adults showed this specific pattern of frequency related BOLD increase, it would be interesting to see if this was predictive of some kind of visual processing behavioral outcome (which might help understand if visual deficits are happening further down the processing stream).

**Response: We have the same research interest. We added a discussion about the above comments to the last paragraph in the Discussion.**

**Responses to Reviewer #2:**

The article by Uchiyama and colleagues submitted for consideration to PLOS One examined BOLD signal changes in older (but not younger) adults in visual cortex in response to flickering checkerboard stimuli, the frequencies of which were parametrically varied between 2, 4, and 8 Hz. Results indicated that, contrary to results reported in Cliff et al. (2013), BOLD signal in fact increases with increasing flicker frequency in older adults, in similar fashion to the pattern widely observed in younger adults, and found no part of visual cortex where BOLD signal at 2 Hz was higher than at 8 Hz.

Overall, the article is well-written, though it may benefit from language editing in a few select places (Enago did a reasonably good job, but it can be improved further). I do have several concerns that I believe, if addressed, will significantly strengthen the manuscript.

Comment 2-1: The central premise of the article seems to be motivated by a single 2013 study by Cliff et al. that showed "decreasing BOLD signal" with increasing flicker frequency. Because it plays so prominently in the rationale behind the study's conception, I would request that the reasoning be more thoroughly fleshed out.

**Response: The rationale has been elaborated by adding the explanation about BOLD signal decrease in the 3^rd^ paragraph of the Introduction.**

Comment 2-2: The Cliff et al. (2013) study does not actually show decreasing BOLD signal in older adults with increasing checkerboard flicker frequency, despite how Figure 4 from that article frames the result. The actual quantity depicted is something called the SSQRatio, which is a ratio of sums of squares, and thus does not account for degrees of freedom. This makes interpretation of any differences in the quantity much less straightforward than those of a traditional/parametric test statistic, and in fact, the authors themselves refer to the statistic itself as a goodness-of-fit statistic. Additionally, while the Cliff et al. article does mention that a significant interaction effect between age group and presentation rate was observed for the SSQRatio in bilateral primary visual cortex, it curiously does not provide any statistical metadata regarding that result.

Other concerns with how the authors from the Cliff et al. article processed the imaging data seem to be sufficient to me to question the result (taken together with the first and second points above). Rather than substantially rewriting the discussion or spending unwarranted manuscript real estate to denigrate another article, I would encourage the authors to reframe the introduction to be less dependent upon that article in motivating the study. I believe the authors of the present article got the treatment of Cliff et al. right in the Discussion section, where it is far less featured.

**Response: The Introduction was edited to be less dependent on Cliff et al. by revising the 2^nd^– 4^th^ paragraph in the Introduction.**

Comment 2-3: I am also curious as to the authors' choice of sample for the study. The interest was very obviously in potential effects of age on the BOLD signal in response to visual stimuli, but knowing that the Cliff et al. study could serve as a (however imperfect) model, why were younger participants not sampled as well? This is not a fatal flaw in design; far from it. But as no data for younger adults are presented here, I would ask the authors to either remove or significantly reword the last sentence from the abstract, as without a younger group, the question of how age affects V1 response is something this study cannot address. The Discussion paragraph that speaks to this (lines 287-292) begins in more appropriately worded fashion, although the last sentence should probably be softened as well, perhaps even by changing "almost the same" to "similar".

**Response: We did not perform the experiment on younger participants. Thus, the last sentences of Abstract and the fifth paragraph of Discussion were misleading for readers. We removed the last sentence of Abstract and revised the sentences of Discussion.**

Comment 2-4: The Materials and Methods section mentions convolution of a boxcar function with a hemodynamic response function. I would request the authors to be more specific here. Which one? Gamma? Double-gamma (similar to the SPM "canonical"? The choice of HRF is very important, as pointed out in Lindquist et al. (2009), especially knowing that older adults were scanned. See West et al. (2019) for concerns regarding changes with age to the BOLD-HRF.

**Response: Thank you for pointing this out. fMRI data were analyzed using a canonical SPM HRF without derivatives. The sentence was added on the Individual analysis section in Materials and Methods as follows:**

**“… (HRF), which was SPM canonical without derivatives.”**

Comment 2-5: The Materials and Methods section mentions that FreeSurfer/FsFast was used for neuroimaging data analysis, but I was not clear whether this was used for post-hoc statistical testing as well. I would request the authors to be more specific here.

**Response: Thank you for pointing this out. Matlab and R were used for post-hoc statistical testing. That was specified in the regions of interest (ROI) analysis section in Materials and methods.**

Comment 2-6: Additional opportunities for language editing did appear from time to time as I read through this manuscript. For example, the first sentence of the ROI analysis subheading of the Materials and Methods section (line 158), sounds more correct as: "To analyze overall V1 activity, ROI analyses were conducted." As I said above, minor changes, but they could be impactful nonetheless.

**Response: Thank you for pointing this out. I have corrected the sentence.**

Comment 2-7: The authors' reporting of statistical test outcomes in the Results section does not conform to the "SAMPL" guidelines listed in PLOS One's author guidance. Per those SAMPL guidelines, "Avoid relying solely on statistical hypothesis testing, such as P values, which fail to convey important information about effect size... P values are not sufficient for re-analysis. Needed instead are descriptive statistics for the variable being compared, including sample size of the groups involved, the estimate (or “effect size”) associated with the P value, and a measure of precision for the estimate, usually a 95% confidence interval." In reading through the Results section, the authors have indeed provided only p-values. I would request that they provide estimates of effect size and, as their chosen alpha was 0.05, 95% confidence intervals.

**Response: As the reviewer has pointed out, data for meta-analysis should be provided in our manuscript. According to the SAMPL guidelines, we have added effect sizes and 95% confidence intervals to Results. Furthermore, the calculation method of correlation coefficient (i.e., Pearson’s product-moment correlation coefficient) has been added in the ROI analysis.**

Comment 2-8: Per the SAMPL guidelines, p-values should not be reported as inequalities. It seems to me as though this is done throughout as a reminder when a significant result is disclosed that it is, indeed, significant, but this is unnecessary and potentially confusing. I would request they remove mentions of "< 0.05".

**Response: “< 0.05” has been removed, followed by p-values in the Results section.**

Comment 2-9: The SAMPL guidelines are perfectly clear as to the use of “NS”: it is to be avoided. As the authors provided the actual p-value in the results (p = 0.054), I would request they simply remove the “NS” from the figure and its mention from the caption.

**Response: The “NS” from Fig 2. and its caption has been removed.**

Comment 2-10: As to the limitation mentioned immediately prior to the Conclusion (lines 293-300), were participants monitored during scanning? If so, this could be mentioned as a point to help offset the potential concern of this limitation for readers.

**Response: The statements that explain that the experimenter monitored the participants’ eyes and that the limitation is a potential concern have been added in the 7^th^ paragraph of Discussion.**
