# Supplementary material for: BOLD signal response in primary visual cortex to flickering checkerboard increases with stimulus temporal frequency in older adults
Source: PLoS One. 2021 Nov 4;16(11):e0259243. doi: 10.1371/journal.pone.0259243 (PMC8568270; doi:10.1371/journal.pone.0259243)
Supplement: Attachment — Submitted filename: Response_to_Reviewers20210909.docx [file pone.0259243.s005.docx]

**Response to Reviewers**

**We thank the editorial board and reviewers for their helpful comments. Our manuscript has been revised according to the review comments. We think that the comments have improved our manuscript. Our point-to-point responses to each comment are written in bold letters.**

**Responses to Reviewer #2:**

The resubmitted article by Uchiyama and colleagues examined BOLD signal changes in older (but not younger) adults in visual cortex in response to flickering checkerboard stimuli, the frequencies of which were parametrically varied between 2, 4, and 8 Hz. Results indicated that, contrary to results reported in Cliff et al. (2013), BOLD signal in fact increases with increasing flicker frequency in older adults, in similar fashion to the pattern widely observed in younger adults, and found no part of visual cortex where BOLD signal at 2 Hz was higher than at 8 Hz.

The authors, by and large, did an excellent job in responding to the concerns I raised in my initial review: the study is more appropriately motivated without undue reliance on the Cliff et al. result, methods were made more detailed, and results were brought in line with SAMPL guidelines. A few manageable but very important concerns remain, however, and I believe the manuscript will be acceptable for publication if these can be addressed.

Comment 2-1: Regarding the choice of the HRF (previous comment 2-4), one critique that remains is the use of a canonical HRF in older populations. Lindquist et al. (2009) elegantly make the point that the choice of HRF used to model measured BOLD signal has important consequences for results and their interpretation. The authors chose the canonical HRF from SPM, a double-gamma function widely employed in the analysis of fMRI data. However, as pointed out in West et al. (2019), this function makes rigid assumptions regarding evolution of the signal’s time-course (e.g., the canonical HRF’s time-to-peak is not free to vary, despite the finding in that study that it is significantly greater in older than in younger adults). This is especially true given that temporal and dispersion derivatives were not included. To be clear, I am not suggesting the authors reprocess their data an additional time using a different HRF selection (though I would consider it to be one way to resolve this criticism). However, in the absence of that step, I do believe that this should be discussed as an additional limitation (in the Discussion section) in reasonable detail for the rationale expounded above.

N.B. My apologies to the authors, in looking at my previous review, the citations I mentioned in comment 2-4 were cut off from the end of the review, and so I have ensured that they now appear in this version.

Lindquist, M. A., Loh, J. M., Atlas, L. Y., & Wager, T. D. (2009). Modeling the hemodynamic response function in fMRI: efficiency, bias and mis-modeling. NeuroImage, 45(1), S187-S198.

West, K. L., Zuppichini, M. D., Turner, M. P., Sivakolundu, D. K., Zhao, Y., Abdelkarim, D., ... & Rypma, B. (2019). BOLD hemodynamic response function changes significantly with healthy aging. NeuroImage, 188, 198-207.

**Response:**

**Thank you for the comments concerning our study limitation. A limitation has been added to the seventh paragraph in the Discussion according to the Comment 2-1, and the references have been cited.**

Comment 2-2: In the Introduction, the beginning the new third paragraph (beginning “The difficulty of seeing brief…”) ostensibly frames the expectation that neural responses in aged primary visual cortex, by way of reduced information processing capability, actually decrease with increasing temporal frequency. This is later confirmed explicitly in the next paragraph. While I believe this to be a reasonable and logical hypothesis based on what is presented in the Introduction, I was surprised to see no Discussion real estate whatsoever expended to explain the fact that the study finds the complete opposite. If the hypothesis was that BOLD signal will decrease monotonically with increasing checkerboard frequency, what would explain the observation that the opposite is actually true? I believe the authors should discuss this as best they can based on the analysis and methodology they used (and in the context of other work - I understand and applaud not wanting to speculate beyond data they observed).

**Response:**

**Thank you for this constructive comment. A paragraph has been added for a discussion on the comment at the end of the Discussion section.**

Comment 2-3: The authors have expanded on the rationale for utilizing surface-based analysis methods over volumetric methods. I agree with this rationale, and would suggest citing more literature (than just Brodoehl et al., 2020) to have an empirical basis upon which to base the expectation that surface-based methods will be superior to volumetric methods used in prior work. Specifically, I would like to see the citations below included.

Hutchison, J. L., Hubbard, N. A., Brigante, R. M., Turner, M., Sandoval, T. I., Hillis, G. A. J., ... & Rypma, B. (2014). The efficiency of fMRI region of interest analysis methods for detecting group differences. Journal of neuroscience methods, 226, 57-65.

Tucholka, A., Fritsch, V., Poline, J. B., & Thirion, B. (2012). An empirical comparison of surface-based and volume-based group studies in neuroimaging. NeuroImage, 63(3), 1443-1453.

**Response:**

**Thank you for this suggestion of literature. The sixth paragraph in the Introduction has been improved, and the suggested references have been cited.**

Comment 2-4: I found the description of the Visual processing speed/UFOV task to be lacking. The authors cited a 2017 article which does describe the task very well, which is fine, but I couldn’t quite discern what the second sentence was saying in the context of this manuscript. I don’t expect the authors to describe the task to the same extent as the 2017 paper, but a little more elaboration/clarification would be appreciated here.

**Response:**

**The section of “Visual processing speed task” has been elaborated to fit the context of this manuscript.**

Comment 2-5: Finding negative BOLD signal relationships with the offset contrast (i.e., the general/frequency-independent effect of stimulus) with no such negative relationships with the slope contrast was interesting. The manuscript indicates that it was in line with previous studies, and cites [43-46], but no such entries appear in the References section. Please add these. Beyond that, though, I felt the (sixth) Discussion paragraph that asserts this as evidence for an experiment and analyses that were well-conducted was lacking. I feel that readers may not follow exactly why this result reinforces the experimental and analytical appropriateness. Please elaborate here (e.g., spatial nature of V1? Anterior/posterior?). I would also like to echo an earlier point – the finding of absolutely no significant clusters that showed a negative relationship to the slope contrast goes completely against the hypothesis laid out in the Introduction, so substantive discussion of one or more potential reasons is warranted.

**Response:**

**Thank you for these comments. The missing references have been added, and the sixth paragraph in the Discussion section has been elaborated. The revised reference numbers are 45–48. Discussion about no significant region with the negative slope was described in the last paragraph in the Discussion section.**

Comment 2-6: Table 3 could use a little cleaning up visually (e.g., the word “Positive” is misspelled, the cells are only merged across columns within rows but gray row dividers are still visible).

**Response:**

**Thank you for finding the problems of the tables. The misspelled word in Table 3 has been corrected, and the gray row lines from all tables have been cleared.**
